# Clinical Characteristics associated with functional seizures in individuals with psychosis

**DOI:** 10.1016/j.schres.2025.05.011

**Published:** 2025-05-20

**Authors:** Allison M. Lake, India A. Reddy, Robert Havranek, Lea K. Davis, Jonah Fox

**Affiliations:** a Vanderbilt Genetics Institute, Vanderbilt University Medical Center, Nashville, TN, USA; b Division of Genetic Medicine, Department of Medicine, Vanderbilt University Medical Center, Nashville, TN, USA; c Department of Psychiatry & Behavioral Sciences, Vanderbilt University Medical Center, Nashville, TN, USA; d Department of Neurology, Vanderbilt University Medical Center, Nashville, TN, USA; e Division of Data-Driven and Digital Medicine, Department of Medicine, Icahn School of Medicine at Mount Sinai, New York, NY, USA

**Keywords:** Functional seizures, Psychosis, Comorbidities, Healthcare utilization

## Abstract

**Background and hypothesis:**

Functional seizures (FS) are episodes characterized by seizure-like events that are not caused by hypersynchronous neuronal activity. Prior studies have suggested an increased prevalence of psychotic disorders among patients with FS, but results have been inconsistent. We hypothesize that FS are associated with psychosis and that among patients with psychosis, the presence of FS may influence patient clinical characteristics, mortality, and medical resource utilization.

**Study design:**

The association between FS and psychosis was assessed using electronic health records data from a total of 761,848 individuals receiving care at Vanderbilt University Medical Center between 1989 and 2023. Analyses of the association between FS and psychiatric outcomes, sexual trauma, healthcare utilization, and other clinical comorbidities were conducted in a subset of 5219 patients with psychosis.

**Study results:**

Odds of FS were elevated among patients with psychosis compared to controls (OR = 10.09, 95 % CI = 8.40–12.13). Among patients with psychosis, those with FS exhibited higher rates of suicidality (OR = 2.18 95 % CI = 1.50–3.17), catatonia (OR = 2.15, 95 % CI = 1.33–3.45), sexual trauma history (OR = 2.93, 95 % CI = 2.00–4.29) and had a greater number of antipsychotic trials (4.63 versus 3.37, beta = 1.23, SE = 0.18, adjusted *p* < 0.001) than those without FS. Furthermore, patients with comorbid FS had more hospital presentations at one, three, five, and ten years after receiving a psychosis diagnosis (adjusted p < 0.001).

**Conclusions:**

FS are more common among patients with psychosis and are associated with increased healthcare utilization as well as an increased prevalence of suicidality, catatonia, and certain psychiatric and medical comorbidities.

## 1. Introduction

Functional seizures (FS) are paroxysmal events of involuntary altered behavior, awareness and/or responsiveness that resemble epileptic seizures (ES) but are not caused by hypersynchronous neuronal activity (Popkirov et al., 2019). The DSM-5 classifies FS as a form of functional neurological disorder (FND) or conversion disorder (American Psychiatric Association, 2013). Patients with FS have an elevated tendency for somatization and dissociation, as well as pathological emotional regulation, attention, and arousal which may be involved in the pathogenesis of FS (Brown and Reuber, 2016). While several explanatory models have been proposed, the etiology of FS remains relatively poorly understood (Brown and Reuber, 2016). FS are more common in women, and the average age of onset is in the second and third decades of life (Bompaire et al., 2021). The gold standard for diagnosing FS and distinguishing it from ES is by recording habitual events with video electroencephalography (EEG) monitoring in an epilepsy monitoring unit (LaFrance Jr et al., 2013). Misdiagnosis is common, and the delay from symptom onset to definitive diagnosis ranges between 3 and 8.4 years (Bompaire et al., 2021). FS does not respond to medications that are used to treat epilepsy but can improve with psychotherapy (Lopez and LaFrance, 2022). FS is associated with significant direct and indirect healthcare costs including frequent emergency department presentations, hospitalizations, and loss of employment (Stephen et al., 2021). Patients with FS have a heightened risk of early death similar to that of patients with ES, which at least in part appears to be explained by the associated comorbidities (Nightscales et al., 2020; Tan et al., 2023).

Post-traumatic stress disorder (PTSD), depression, anxiety, chronic pain, insomnia, migraine, and asthma are among the more commonly recognized comorbidities in patients with FS (Popkirov et al., 2019). Other comorbidities such as psychotic disorders and opioid use were found to explain the increased morality risk found among patients with FS (Tan et al., 2023). A few prior studies suggested that psychotic disorders have an elevated prevalence among patients with FS, but the findings were inconsistent, with a meta-analysis finding that the reported prevalence of psychosis ranged from 0 to 15 % among those with FS (Diprose et al., 2016). It is unclear whether the prevalence of FS is elevated among patients with psychosis, but there are reasons to suspect that it may be. For instance, schizophrenia patients demonstrate higher rates of dissociative and functional symptoms when compared to healthy controls (Kanchanatawan et al., 2017; Schäfer et al., 2012). In addition, traumatic experiences, which are strongly associated with FS, are significantly more common among patients with psychosis compared to the general population (Popkirov et al., 2019; Martin et al., 2023; Hardy and Mueser, 2017). A systematic review reported that 78.9 % of studies found a prevalence of PTSD exceeding 10 % among patients with schizophrenia, which is significantly higher than the general population (Dallel et al., 2018). There is also some evidence to suggest that patients with psychosis and adverse childhood experiences have worse outcomes including poorer health and increased healthcare resource utilization. (Rosenberg et al., 2007; Trotta et al., 2015; Chartier et al., 2010) Therefore, we hypothesize that the prevalence of FS is elevated among patients with psychosis and that FS may also impact comorbidities, healthcare resource utilization, psychiatric outcomes, treatment, and mortality in this patient population.

## 2. Methods

### 2.1. Phenotype definitions and validation

Clinical data including demographics, diagnosis and procedure codes, medication records, and clinical notes were extracted from the Synthetic Derivative, a de-identified copy of the Vanderbilt University Medical Center (VUMC) electronic medical record. To mitigate nonrandom missingness between cases and controls, a data floor requiring at least five International Classification of Diseases, Ninth Revision (ICD-9) or Tenth Revision (ICD-10) codes of any type documented over the age of 18 on separate days across a period at least three years was applied. Given that sex was included as a covariate in all statistical analyses, a total of ten individuals were excluded from analysis due to missing sex, resulting in a total of 761,848 individuals in the full EHR sample (Table 1). The psychosis phenotype was defined using a curated list of ICD-9 and ICD-10 codes relating to psychosis (eTable 1). A patient was designated as a psychosis case if they had documentation, at age 18 or older, of a psychosis diagnosis code on ≥3 separate calendar months or ≥ 2 separate calendar months plus at least one antipsychotic medication record. Patients with insufficient evidence for a psychosis diagnosis (e.g., only one diagnosis code) or meeting case criteria only before the age of 18 were excluded from the final cohort and all downstream analyses.

The psychosis phenotype definition was validated by manual review of 50 randomly selected patients meeting phenotype criteria. Each patient chart was independently reviewed by a psychiatrist (IAR) and a neurologist (JF) and labeled as having a specific psychotic disorder (schizophrenia; schizoaffective disorder, bipolar type; schizoaffective disorder, depressive type; schizoaffective disorder, unspecified type; bipolar affective disorder with psychotic features, major depressive disorder with psychotic features; delusional disorder; psychosis in other primary psychiatric conditions), having a possible psychotic disorder, or not having a psychotic disorder. Discrepancies between reviewers were resolved through discussion and consensus. Of the 50 patient charts reviewed, 37 (74 %) were labeled as having probable psychosis, while 42 (84 %) were determined to have a probable or possible psychosis diagnosis.

The functional seizure phenotype was based on a previously validated algorithm which correctly identified 98 % of cases as likely or possible FS in the VUMC health record (Goleva et al., 2020). Briefly, cases were defined as having a diagnosis code for convulsions or conversion disorder, the presence of an FS keyword and the keyword “EEG” in at least one clinical note, and the presence of an EEG procedure code, indicating that the patient was evaluated for seizure activity. Patients with some evidence of FS but not enough evidence to be classified as a case (e.g., presence of a convulsions code but not an FS keyword; see Goleva et al.) or meeting case criteria only before the age of 18, were excluded from the final cohort and all downstream analyses. Disclosures of sexual trauma were extracted from clinical notes using matches to key words and phrases from a phenotyping algorithm previously validated at VUMC (algorithm version 2, available at https://phekb.org/phenotype/sexual-assault-disclosures-clinical-notes-v2) (Lake et al., 2023). Remaining individuals were designated as controls. Patients with specific diagnosis codes for generalized or focal epilepsy (listed in eTable 1) were excluded from the control group for all analyses. As functional seizures often co-occur with epileptic seizures in the same individuals, we conducted our association analyses in the full EHR sample using two FS definitions: one with concurrent epilepsy cases included, and one with these cases excluded. In subsequent analyses in the psychosis cohort (*N* = 5219), patients with concurrent focal or generalized epilepsy codes were excluded from both cases and controls, due to the concern that inclusion of FS cases with concurrent epilepsy may introduce confounding. This is because epilepsy is also associated with elevated rates of psychiatric and other comorbidities, and thus statistical associations with clinical phenotypes may be driven by epilepsy, rather than FS.

Additional psychiatric outcomes including diagnoses of catatonia or suicidal behavior, electroconvulsive therapy (ECT) procedures (Current Procedural Terminology code 90870), and antipsychotic medication trials were extracted using ICD-9 and ICD-10 codes, procedure codes, and medication records, respectively. A full list of these codes can be found in eTable 1. For the phenome-wide association analysis (PheWAS), related diagnostic codes were grouped into “phecodes” using the R PheWAS package, requiring two component codes on distinct dates for each diagnosis (Denny et al., 2010; Carroll et al., 2014; Wu et al., 2019).

Information on inpatient and emergency department (ED) encounters on or after the earliest psychosis diagnosis was extracted for the psychosis cohort healthcare utilization analysis using a combination of visit records and procedure codes. To avoid double-counting encounters, inpatient encounters occurring within three days of one another were combined into a single encounter, and ED encounters within one day of one another were combined into a single encounter. The total number of ED and inpatient encounters for each patient in the psychosis cohort were counted at one, three, five, and ten years from the earliest psychosis diagnosis.

A subset of 100 patients with psychosis and at least one encounter record within one year of the earliest psychosis diagnosis (50 with co-morbid FS, 50 with no FS) were randomly sampled, and clinical notes from all encounters within the first year of the earliest psychosis diagnosis were extracted. Notes were manually reviewed by neurologists JF and RH and medical student AML. Based on clinical note documentation, the diagnostic category of the presenting problem for each encounter was determined. For the purposes of this chart review, a patient was considered to have definite FS if their habitual events were captured by video-EEG recording. If the clinical description of patient events were suspicious for FS and there was no evidence of epileptiform abnormalities on EEG the patient was considered to have suspected FS. This research study was reviewed and approved by the VUMC IRB (IRB#160650) and received a “non-human subjects” determination due to the use of deidentified medical record data.

### 2.2. Statistical analysis

Statistical analyses were conducted in R version 4.2.1 (R: A language and environment for statistical computing. Version 4.2.1. Vienna, Austria, 2022). All regression models included covariates for EHR-recorded sex and either age at earliest psychosis diagnosis (temporally restricted psychosis cohort analyses) or median age at visit (non-temporally restricted analyses). The associations between FS and psychiatric diagnoses were tested using logistic regression in the full EHR sample. Further analyses were conducted in the psychosis patient cohort (*N* = 5219). Differences between psychosis patient groups in outcomes at any EHR time point were assessed using logistic regression for dichotomous outcomes (clozapine and ECT treatment, catatonia diagnoses, codes for suicidal ideation or attempt) or linear regression for continuous outcomes (number of unique antipsychotic medications trialed). PheWAS was performed on 578 phecodes for which there were at least 50 cases total, with at least 5 for each sex (to avoid inclusion of sex-specific phecodes). Differences in numbers of encounters at one, three, five, and ten years after the earliest psychosis diagnosis were assessed using Poisson regression with covariates for sex and age at psychosis diagnosis. In the encounter chart review analysis, differences in presenting problems between groups were assessed in separate logistic regressions for each diagnosis category. Time-to-event analysis was performed using Cox proportional hazards regression, and survival curves were generated using the *survival* and *survminer* R packages, respectively (Therneau, n.d.; Therneau and Grambsch, 2013; *survminer: Drawing Survival Curves using ’ggplot*2, 2021). All statistically significant results reported in the text passed multiple testing correction (maximum *p* < 0.05) using the Bonferroni method, adjusting for the total number of tests in each specific analysis.

## 3. Results

Analyses were conducted using EHR data from a total of 761,848 individuals receiving care at VUMC between 1989 and 2023, among whom 5290 individuals had a diagnosis of psychosis and 3675 were identified as FS cases (1678 with concurrent generalized or focal epilepsy diagnoses, 1997 without, Table 1). Psychosis diagnoses were more common in patients with FS (6.3 % among those excluding concurrent epilepsy, 4.2 % among those including concurrent epilepsy) than in FS controls (0.7 %, Table 1). FS case status was significantly associated with diagnoses of psychosis in the VUMC cohort, with slightly stronger associations when excluding concurrent epilepsy from cases (OR = 10.09, 95 % CI = 8.40–12.13) than when including these individuals (OR = 8.45, 95 % CI = 7.29–9.79, Table 2). For comparison, we quantified associations between FS and other mental health conditions (depression and FS without concurrent epilepsy, OR = 7.25, 95 % CI = 6.57–7.99; post-traumatic stress disorder and FS without concurrent epilepsy, OR = 17.97, 95 % CI = 16.04–20.14; Table 2). All associations between FS and psychiatric diagnoses were statistically significant (*p* < 0.001) after conservative Bonferroni correction for 6 total tests.

Subsequent analyses focused on 5219 patients with psychosis and excluded those with epilepsy diagnoses to disambiguate FS from epilepsy within the psychosis cohort. Among patients with a psychosis diagnosis, 67.5 % of those with comorbid FS (*N* = 126) were female, as compared with 51.1 % of those without FS (χ^2^ = 12.45, p < 0.001, Table 3). Of the 126 patients in the comorbid group, 57 (45.2 %) had evidence of FS (diagnosis code or clinical note with a relevant keyword) before the first psychosis diagnosis code. Psychosis patients with co-morbid FS were more likely to receive a diagnostic code for suicidal ideation, suicide attempt, or self-harm (OR = 2.18 95 % CI = 1.50–3.17) and had a greater total number of antipsychotic trials on average (4.63 versus 3.37, beta = 1.23, SE = 0.18, Table 4). Patients with comorbid FS also had a significantly greater odds of having a catatonia diagnosis (OR = 2.15, 95 % CI = 1.33–3.45) and a history of sexual trauma (OR = 2.93, 95 % CI = 2.00–4.29, Table 4) than those without FS. No differences in odds of receiving ECT or clozapine treatment were observed between patient groups.

**Fig. 1.**
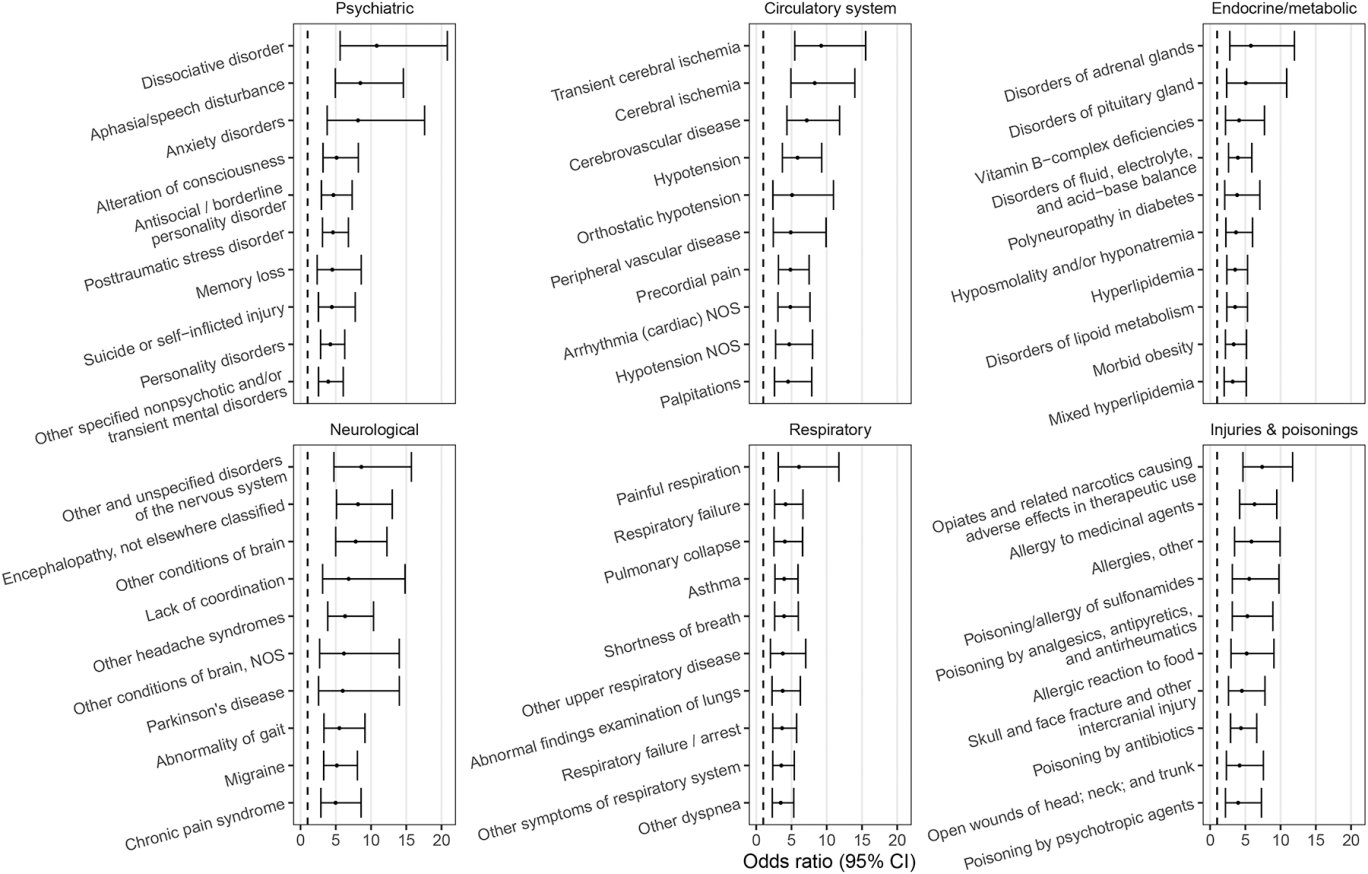
Top ten clinical comorbidities per broad diagnostic category associated with functional seizures among patients with a psychosis diagnosis. The top 10 largest-effect associations among phenotypes passing multiple testing correction (p < 8.65e-05, Bonferroni-adjusted for 578 phenotypes tested) are shown per diagnostic category. All regressions were adjusted for EHR-recorded sex and EHR-median age. Ten neuropsychiatric phenotypes directly related to functional seizures (somatoform disorder, convulsions, altered mental status, etc.) were removed for clarity. Abbreviations: NOS, not otherwise specified.

Differences in comorbidities between patient groups were assessed using PheWAS. A total of 156 phenotypes were significantly associated with FS among patients with psychosis (*p* < 8.65e-05, Bonferroni-corrected for 578 phenotypes tested). Diagnostic categories with the largest number of associated phenotypes included circulatory, neurological, psychiatric, endocrine, and respiratory conditions, as well as injuries and poisonings (Fig. 1).

We examined differences in numbers of emergency department (ED) and inpatient encounters between patient groups at time intervals relative to the index psychosis diagnosis. Patients with comorbid FS had significantly greater numbers of inpatient and ED encounters at one, three, five, and ten years after the index psychosis diagnosis (*p* < 0.001, Bonferroni-corrected for eight tests, Table 5).

Clinical notes from a subset of 100 randomly selected individuals (50 with comorbid FS, 50 with psychosis only) were reviewed, and the primary reasons for ED presentations or inpatient hospitalizations within the first year of receiving a psychosis diagnosis were assessed. Across patient groups, the most common reasons for presentation (≥10 patients total) were psychiatric symptoms, injuries and poisonings, circulatory problems, respiratory problems, musculoskeletal problems, neurological symptoms, and digestive problems. No differences in the odds of any diagnostic category or of suicidal ideation or attempt were observed between patient groups (eTable 2, eTable 3). Of the 50 patients with comorbid FS, 24 % were determined to have likely or definite functional seizure symptoms as part of their clinical presentation (eTable 3).

Survival analysis indicated no significant differences between patient groups in mortality after the earliest psychosis diagnosis in the full sample (hazard ratio = 1.45, 95 % CI = 0.93–2.27, eFigure 1). Given higher mortality rates observed in men, we repeated the analysis stratified by sex and found no significant difference in either group (females, hazard ratio = 1.29, 95 % CI = 0.73–2.30; males, hazard ratio = 1.80, 95 % CI = 0.89–3.63; eFigure 2). Given that 16 of the 126 individuals (12.7 %) with comorbid FS were first documented to have FS five years or more after the date of their first psychosis diagnosis, to mitigate immortal time bias, we conducted an additional analysis in the sex-combined sample removing these individuals and found similar results (hazard ratio = 1.50, 95 % CI = 0.93–2.44).

## 4. Discussion

The results of our study suggest that FS is not only more common among patients with psychosis but that its presence may have significant clinical and medical resource utilization implications. Comorbid FS was associated with an increase in inpatient and emergency department presentations, documented antipsychotic trials, catatonia diagnoses, and suicidal ideation or attempts. Furthermore, psychosis patients with comorbid FS were more likely to have a history of sexual trauma than those without FS. Finally, psychosis patients with comorbid FS had significantly different comorbidity profiles.

Psychosis is associated with substantial medical expenditures and healthcare utilization. In the United States, the annual cost attributable to schizophrenia was estimated to be 155.7 billion dollars (Cloutier et al., 2016). Schizophrenia is a predictor of increased emergency department utilization for both psychiatric and medical disorders and increases likelihood of admission (Ronaldson et al., 2020). FS is also associated with considerable medical resource utilization including frequent emergency department presentations (Stephen et al., 2021). Our data suggests that the presence of both disorders increases emergency department presentations, hospital admissions, and number of antipsychotic trials compared to psychosis alone.

In a significant proportion of the manually reviewed hospital presentations, the reasons for which psychosis patients—both with and without comorbid FS—presented to the hospital were not directly related to either diagnosis but to other medical or psychiatric indications. Prior research has indicated that a significant proportion of the increased healthcare utilization among both FS and psychosis patients are for the treatment of comorbid conditions (Ronaldson et al., 2020; Ramamurthy et al., 2021; Salinsky et al., 2016). Poor adherence to treatment and inadequate access to outpatient care may account for why some patients may utilize the emergency department (Hardy et al., 2018), while adequate treatment of psychosis may potentially reduce healthcare utilization (Okoli et al., 2022). Similarly, healthcare utilization significantly declines following both the diagnosis and treatment of FS (Salinsky et al., 2016; Deleuran et al., 2019). This is notable given that patients with FS often wait years from the time of symptom onset to receiving an accurate diagnosis (Bompaire et al., 2021). However, we did not evaluate the relationship between healthcare utilization and the timing of FS diagnosis or treatment in this study.

Adverse childhood experiences, which may include physical and sexual abuse. have been associated with an increased risk for developing psychosis, an earlier onset of schizophrenia, suicidal behavior, and FS (Popkirov et al., 2019; Goleva et al., 2020; Berardelli et al., 2021). We found that psychosis patients with comorbid FS were more likely to have a history of sexual trauma than those without FS. This shared risk factor may partially explain the associations between FS, psychosis, and suicidality (Yates et al., 2019; Chapman et al., 2015; Gupta et al., 2020). Psychosis is well established as a significant risk factor for suicide (Girgis, 2020), and our results suggest that FS may be associated with a further elevated risk for suicidal ideation or attempts.

Comorbid FS was associated with additional antipsychotic medication trials but no other markers indicative of psychosis severity or treatment responsiveness such as clozapine prescriptions or ECT treatment. Patients with FS may have had more medication trials because they have an increased tendency to report medication allergies, and those with somatization are more likely to report medication intolerances (Robbins et al., 2016; Hassel et al., 2011). Catatonia diagnoses were significantly more common in patients with comorbid FS. Prior work has found that there may be an association between psychological trauma and catatonia which may account for this finding (Ross and Browning, 2016; Biles et al., 2021).

There was no significant difference in the primary reason for emergency department presentations and inpatient admissions between the groups among the chart reviewed subset, but the two patient groups did exhibit significantly different medical comorbidity profiles. For instance, patients with comorbid FS were more likely to also have migraine, chronic pain, certain types of respiratory problems such as asthma, allergies, hyperlipidemia, and vascular disease. These conditions have previously been associated with FS (Popkirov et al., 2019; Robbins et al., 2016; Fox et al., 2022; Fox and Mishra, 2024). Psychosis has also been linked to higher prevalences of vascular risk factors and disease (Li et al., 2023; Galletly et al., 2012). Chronic pain has a similar prevalence in patients with schizophrenia and the general population, but it is speculated that it may be underdiagnosed due to differences in pain perception and under-reporting (Stubbs et al., 2014).

Both psychosis and FS are associated with an elevated risk of mortality (Nightscales et al., 2020; Tan et al., 2023; Oakley et al., 2018). Our results suggested that the mortality risk of psychosis patients was not significantly different based on the presence or absence of comorbid FS. Given that the elevated mortality risk in FS appears to be driven by associated comorbidities and that psychosis is also associated with mortality there may be no significant added risk when both conditions are present (Tan et al., 2023). On the other hand, it is possible that we had an insufficient sample size to detect a difference and/or that there is a difference in certain subgroups. We examined survival curves stratified by sex and found that neither male nor female psychosis patients with FS had an increased mortality risk.

Limitations of this study include the use of secondary EHR data to identify patients and the retrospective design of the study. The algorithms used to identify patients with psychosis and FS were validated and found to have an adequate positive predictive value, with multiple lines of EHR evidence required for FS and psychosis diagnoses, including diagnosis codes, medications, procedure codes, and clinical notes. It is therefore likely that at some point in their interactions with the health care system, FS and psychosis cases were evaluated by a psychiatrist and/or neurologist. However, we acknowledge that all EHR-based phenotyping algorithms are limited by missing information and imprecision of diagnosis codes and that our FS algorithm was not validated specifically within psychosis patients. Further, the sample size of patients with psychosis and comorbid FS was relatively small, which may have limited our sensitivity to detect certain findings. Our mortality analysis was limited by the information available in the EHR, and it is probable that missing data contributed to the null results of this analysis. Nonetheless, the limitations diagnostic imprecision (in particular, the potential for the presence of true cases in the control group), and limited sample size are expected to bias associations towards the null. Thus, these limitations do not decrease our confidence in the significant associations that were detectable in these data.

## 5. Conclusion

Psychosis patients have an increased prevalence of FS, which is associated with unique clinical characteristics including an elevated use of medical resources, suicidality, and certain comorbidities. Increased awareness and screening of psychosis patients for comorbid FS may assist in the facilitation of optimal care. Future research could evaluate how treatment of either disorder affects outcomes in this unique group of patients.

## Supplementary Material

eTable 2

eTable 1

Supplementary data to this article can be found online at https://doi.org/10.1016/j.schres.2025.05.011.

## Figures and Tables

**Table 1:** Sample characteristics.

	FS excluding epilepsy^a^ (*N* = 1997)	FS including epilepsy (*N* = 1678)	Controls (*N* = 758,173)
EHR-recorded sex			
Female	1480 (74.1 %)	1201 (71.6 %)	457,568 (60.4 %)
Male	517 (25.9 %)	477 (28.4 %)	300,605 (39.6 %)
Median age at encounter (years)			
Mean (SD)	42.3 (14.4)	42.2 (14.7)	50.4 (18.1)
Median (Min, Max)	41.4 (6.9, 88.5)	41.9 (9.5, 88.0)	50.9 (0.0, 90.0)
EHR-recorded race			
Black	242 (12.1 %)	237 (14.1 %)	84,598 (11.2 %)
White	1705 (85.4 %)	1398 (83.3 %)	611,753 (80.7 %)
Other or unknown	50 (2.5 %)	43 (2.6 %)	61,822 (8.2 %)
Total no. encounters			
Mean (SD)	81.6 (127.9)	92.8 (127.7)	43.1 (60.9)
Median (Min, Max)	36.0 (5.0, 1614.0)	50.0 (5.0, 1692.0)	23.0 (5.0, 2201.0)
Record length (years)			
Mean (SD)	12.9 (6.8)	14.0 (7.2)	11.3 (6.2)
Median (Min, Max)	12.1 (3.0, 32.7)	13.6 (3.0, 32.7)	10.3 (3.0, 32.8)
Psychosis			
Case	126 (6.3 %)	71 (4.2 %)	5093 (0.7 %)
Control	1745 (87.4 %)	1487 (88.6 %)	744,773 (98.2 %)
Missing	126 (6.3 %)	120 (7.2 %)	8307 (1.1 %)
Depression			
Case	730 (36.6 %)	642 (38.3 %)	67,616 (8.9 %)
Control	922 (46.2 %)	788 (47.0 %)	648,634 (85.6 %)
Missing	345 (17.3 %)	248 (14.8 %)	41,923 (5.5 %)
PTSD			
Case	403 (20.2 %)	259 (15.4 %)	9495 (1.3 %)
Control	1464 (73.3 %)	1338 (79.7 %)	743,416 (98.1 %)
Missing	130 (6.5 %)	81 (4.8 %)	5262 (0.7 %)

Abbreviations: EHR, electronic health record; FS, functional seizures; SD, standard deviation; PTSD, post-traumatic stress disorder.

a Patients with specific codes for generalized or focal epilepsy were excluded from FS cases.

**Table 2:** Associations between functional seizures and mental health conditions in the full electronic health record sample.

Diagnosis	FS algorithm	Beta^a^	SE	Statistic	OR (95 % CI)^b^	N
Psychosis (*Validated algorithm)*	Excluding epilepsy	2.31	0.09	24.66	10.09 (8.40–12.13)	751,737
	Including epilepsy	2.13	0.08	28.41	8.45 (7.29–9.79)	753,295
Depression *(Phecode 296.2)*	Excluding epilepsy	1.98	0.05	39.70	7.25 (6.57–7.99)	717,902
	Including epilepsy	2.00	0.04	54.54	7.37 (6.86–7.92)	719,332
PTSD *(Phecode 300.9)*	Excluding epilepsy	2.89	0.06	49.73	17.97 (16.04–20.14)	754,778
	Including epilepsy	2.73	0.05	60.65	15.39 (14.09–16.82)	756,375

Abbreviations: FS, functional seizures; SE, standard error; OR, odds ratio; CI, confidence interval, PTSD, post-traumatic stress disorder.

a Each logistic regression model adjusted for sex and median age at encounter.

b All tests were statistically significant (*p* < 0.001) after Bonferroni correction for 6 total tests.

**Table 3:** Psychosis cohort sample characteristics.

	Comorbid FS^a^ (N = 126)	No FS (*N* = 5093)
EHR-recorded sex		
Female	85 (67.5 %)	2605 (51.1 %)
Male	41 (32.5 %)	2488 (48.9 %)
Median age at encounter (years)		
Mean (SD)	43.7 (14.1)	45.7 (15.6)
Median (Min, Max)	44.1 (18.0, 75.2)	45.8 (11.0, 89.1)
EHR-recorded race		
Black	20 (15.9 %)	1486 (29.2 %)
White	103 (81.7 %)	3393 (66.6 %)
Other / unknown	3 (2.4 %)	214 (4.2 %)
Total no. encounters		
Mean (SD)	181.5 (187.6)	92.1 (111.5)
Median (Min, Max)	121.0 (11.0, 1121.0)	54.0 (5.0, 1393.0)
Record length (years)		
Mean (SD)	16.3 (7.4)	13.8 (6.9)
Median (Min, Max)	16.0 (3.3, 32.7)	13.5 (3.0, 32.5)

Abbreviations: EHR, electronic health record; FS, functional seizures; SD, standard deviation.

a Patients with specific codes for generalized or focal epilepsy were excluded from FS cases.

**Table 4:** Associations between functional seizures and psychiatric outcomes or phenotypes among patients with psychosis.

	No. with phenotype (%) or mean (SD)					
Phenotype	Comorbid FS	No FS	Beta^a,b^	SE	Statistic	OR (95 % CI)
Total antipsychotics	4.63 (2.22)	3.37 (1.95)	1.23**	0.18	7.00	NA
Clozapine	15 (11.9 %)	458 (9.0 %)	0.37	0.28	1.32	1.45 (0.84–2.51)
ECT	15 (11.9 %)	394 (7.7 %)	0.50	0.28	1.77	1.64 (0.95–2.86)
Clozapine or ECT (combined)	25 (19.8 %)	786 (15.4 %)	0.35	0.23	1.53	1.42 (0.91–2.21)
Catatonia diagnosis	22 (17.5 %)	442 (8.7 %)	0.76*	0.24	3.15	2.15 (1.33–3.45)
Suicidal ideation or attempt	81 (64.3 %)	2310 (45.4 %)	0.78**	0.19	4.07	2.18 (1.50–3.17)
Sexual trauma disclosure	48 (38.1 %)	785 (15.4 %)	1.08**	0.19	5.53	2.93 (2.00–4.29)

Abbreviations: FS, functional seizures; ECT, electroconvulsive therapy; SD, standard deviation; SE, standard error; OR, odds ratio; CI, confidence interval.

a All regressions were adjusted for EHR-recorded sex and EHR-median age. All associations were tested using logistic regression, with the exception of the total antipsychotic trials outcome, which was tested using linear regression.

b *P*-values were adjusted for 7 tests using the Bonferroni method. Adjusted p-values are indicated by asterisks (**, p < 0.001; *, p < 0.05).

**Table 5:** Differences in number of emergency department and inpatient encounters between FS groups, among patients with psychosis.

		Mean no. visits				
Visit type	Time point	FS	No FS	Beta^a,b^	SE	Statistic
ED	1 year	2.17	1.12	0.73	0.06	11.68
Inpatient	1 year	1.44	1.02	0.34	0.08	4.52
ED	3 years	4.99	2.11	0.91	0.04	22.20
Inpatient	3 years	2.83	1.53	0.61	0.05	11.29
ED	5 years	7.40	2.83	1.01	0.03	29.66
Inpatient	5 years	3.99	1.87	0.75	0.05	16.42
ED	10 years	10.99	3.93	1.06	0.03	38.09
Inpatient	10 years	5.67	2.37	0.87	0.04	22.48

Abbreviations: ED, emergency department; FS, functional seizures; SE, standard error.

a All regressions were adjusted for EHR-recorded sex and EHR-median age. Associations were tested using Poisson regression.

b All tests were statistically significant (p < 0.001) after Bonferroni correction for 8 total tests.

## References

[R1] American Psychiatric Association, 2013. Diagnostic and Statistical Manual of Mental Disorders: DSM-5 (5th edition). American Psychiatric Association, Washington, DC.

[R2] Berardelli I, Rogante E, Sarubbi S, Erbuto D, Lester D, Pompili M, 2021. The importance of suicide risk formulation in schizophrenia. Front. Psych 12, 779684. 10.3389/fpsyt.2021.779684 PMC8716825 34975579

[R3] Biles TR, Anem G, Youssef NA, 2021. Should catatonia be conceptualized as a pathological response to trauma? J. Nerv. Ment. Dis 209 (5), 320–323. 33835951 10.1097/NMD.0000000000001300

[R4] Bompaire F, Barthelemy S, Monin J, Quirins M, Marion L, Smith C, Boulogne S, Auxemery Y, 2021. PNES epidemiology: what is known, what is new? Eur. J. Trauma Dissociation 5 (1), 100136.

[R5] Brown RJ, Reuber M, 2016. Towards an integrative theory of psychogenic non-epileptic seizures (PNES). Clin. Psychol. Rev 47, 55–70. 27340856 10.1016/j.cpr.2016.06.003

[R6] Carroll RJ, Bastarache L, Denny JC, Aug 15 2014. R PheWAS: data analysis and plotting tools for phenome-wide association studies in the R environment. Bioinformatics 30 (16), 2375–2376. 24733291 10.1093/bioinformatics/btu197 PMC4133579

[R7] Chapman CL, Mullin K, Ryan CJ, Kuffel A, Nielssen O, Large MM, 2015. Meta-analysis of the association between suicidal ideation and later suicide among patients with either a schizophrenia spectrum psychosis or a mood disorder. Acta Psychiatr. Scand 131 (3), 162–173. 10.1111/acps.12359. 25358861

[R8] Chartier MJ, Walker JR, Naimark B, 2010. Separate and cumulative effects of adverse childhood experiences in predicting adult health and health care utilization. Child Abuse Negl. 34 (6), 454–464. 20409586 10.1016/j.chiabu.2009.09.020

[R9] Cloutier M, Aigbogun MS, Guerin A, Nitulescu R, Ramanakumar AV, Kamat SA, DeLucia M, Duffy R, Legacy SN, Henderson C, Francois C, Wu E, 2016. The economic burden of schizophrenia in the United States in 2013. J. Clin. Psychiatry 77 (6), 764–771. 10.4088/JCP.15m10278. 27135986

[R10] Dallel S, Cancel A, Fakra E, 2018. Prevalence of posttraumatic stress disorder in schizophrenia spectrum disorders: a systematic review. Neuropsychiatry 8 (3), 1027–1037.

[R11] Deleuran M, Nørgaard K, Andersen NB, Sabers A, 2019. Psychogenic nonepileptic seizures treated with psychotherapy: long-term outcome on seizures and healthcare utilization. Epilepsy Behav. 98, 195–200, 2019/09/01/. 31377661 10.1016/j.yebeh.2019.05.007

[R12] Denny JC, Ritchie MD, Basford MA, , May 1 2010. PheWAS: demonstrating the feasibility of a phenome-wide scan to discover gene-disease associations. Bioinformatics 26 (9), 1205–1210. 20335276 10.1093/bioinformatics/btq126 PMC2859132

[R13] Diprose W, Sundram F, Menkes DB, 2016. Psychiatric comorbidity in psychogenic nonepileptic seizures compared with epilepsy. Epilepsy Behav. 56, 123–130. 26874243 10.1016/j.yebeh.2015.12.037

[R14] Fox J, Mishra M, 2024. Hypertension and other vascular risk factors in patients with functional seizures. Epilepsy Behav. 152, 109650. 38277850 10.1016/j.yebeh.2024.109650

[R15] Fox J, Goleva SB, Haas KF, Davis LK, Mar 2022. Functional seizures are associated with cerebrovascular disease and functional stroke is more common in patients with functional seizures than epileptic seizures. Epilepsy Behav. 128, 108582. 35123242 10.1016/j.yebeh.2022.108582 PMC8898282

[R16] Galletly CA, Foley DL, Waterreus A, Watts GF, Castle DJ, McGrath JJ, Mackinnon A, Morgan VA, 2012. Cardiometabolic risk factors in people with psychotic disorders: the second Australian national survey of psychosis. Aust. N. Z. J. Psychiatry 46 (8), 753–761. 22761397 10.1177/0004867412453089

[R17] Girgis RR, 2020. The neurobiology of suicide in psychosis: a systematic review. J. Psychopharmacol. 34 (8), 811–819. 32638623 10.1177/0269881120936919

[R18] Goleva SB, Lake AM, Torstenson ES, Haas KF, Davis LK, Dec 1 2020. Epidemiology of functional seizures among adults treated at a university hospital. JAMA Netw. Open 3 (12), e2027920. 33372972 10.1001/jamanetworkopen.2020.27920 PMC7772716

[R19] Gupta R, Garg D, Kumar N, Singh MB, Shukla G, Goyal V, Pandey RM, Srivastava AK, 2020. Psychiatric co-morbidities and factors associated with psychogenic non-epileptic seizures: a case–control study. Seizure 81, 325–331, 2020/10/01/. 32660849 10.1016/j.seizure.2020.05.007

[R20] Hardy KV, Mueser KT, 2017. Editorial: trauma, psychosis and posttraumatic stress disorder. Front. Psych 8, 220. 10.3389/fpsyt.2017.00220 PMC5675853 29163239

[R21] Hardy M, Jackson C, Byrne J, 2018. Antipsychotic adherence and emergency department utilization among patients with schizophrenia. Schizophr. Res 201, 347–351. 29895413 10.1016/j.schres.2018.06.006

[R22] Hassel JC, Danner D, Hassel AJ, Oct 2011. Psychosomatic or allergic symptoms? High levels for somatization in patients with drug intolerance. J. Dermatol 38 (10), 959–965. 21767296 10.1111/j.1346-8138.2011.01249.x

[R23] Kanchanatawan B, Sirivichayakul S, Thika S, , 2017. Physio-somatic symptoms in schizophrenia: association with depression, anxiety, neurocognitive deficits and the tryptophan catabolite pathway. Metab. Brain Dis 32, 1003–1016. 28258445 10.1007/s11011-017-9982-7

[R24] LaFrance WC Jr., Baker GA, Duncan R, Goldstein LH, Reuber M, 2013. Minimum requirements for the diagnosis of psychogenic nonepileptic seizures: a staged approach: a report from the International League Against Epilepsy Nonepileptic Seizures Task Force. Epilepsia 54 (11), 2005–2018. 24111933 10.1111/epi.12356

[R25] Lake AM, Goleva SB, Samuels LR, Carpenter LM, Davis LK, 2023. Sex differences in health conditions associated with sexual assault in a large hospital population. Complex Psychiat. 8 (3–4), 80–89. 10.1159/000527363 PMC10288064 36660008

[R26] Li X, Hu S, Liu P, 2023. Vascular-related biomarkers in psychosis: a systematic review and meta-analysis. Front. Psych 14, 1241422. 10.3389/fpsyt.2023.1241422 PMC10486913 37692299

[R27] Lopez MR, LaFrance WC, 2022. Treatment of psychogenic nonepileptic seizures. Curr. Neurol. Neurosci. Rep 22 (8), 467–474. 10.1007/s11910-022-01209-3. 35674871

[R28] Martin D, Philips M, Greenstone H, Davies J, Stewart G, Ewins E, Zammit S, 2023. Examining the relationship between trauma, post-traumatic stress disorder and psychosis in patients in a UK secondary care service. Psychiatr. Res. Clin. Pract 5 (2), 51–59. Summer. 37293141 10.1176/appi.prcp.20220028 PMC10245461

[R29] Nightscales R, McCartney L, Auvrez C, , 2020. Mortality in patients with psychogenic nonepileptic seizures. Neurology 95 (6), e643–e652. 32690794 10.1212/WNL.0000000000009855

[R30] Oakley P, Kisely S, Baxter A, Harris M, Desoe J, Dziouba A, Siskind D, 2018. Increased mortality among people with schizophrenia and other non-affective psychotic disorders in the community: a systematic review and meta-analysis. J. Psychiatr. Res 102, 245–253. 29723811 10.1016/j.jpsychires.2018.04.019

[R31] Okoli CTC, Kappi A, Wang T, Makowski A, Cooley AT, 2022. The effect of long-acting injectable antipsychotic medications compared with oral antipsychotic medications among people with schizophrenia: a systematic review and meta-analysis. Int. J. Ment. Health Nurs 31 (3), 469–535. 34931437 10.1111/inm.12964

[R32] Popkirov S, Asadi-Pooya AA, Duncan R, , 2019. The aetiology of psychogenic non-epileptic seizures: risk factors and comorbidities. Epileptic Disord. 21 (6), 529–547. 31843732 10.1684/epd.2019.1107

[R33] R: A language and environment for statistical computing. Version 4.2.1. Vienna, Austria, 2022.

[R34] Ramamurthy S, Steven Brown L, Agostini M, , 2021. Emergency department visits and readmissions in patients with psychogenic nonepileptic seizures (PNES) at a safety net hospital. Epilepsy Behav. 122, 108225, 2021/09/01/. 34352667 10.1016/j.yebeh.2021.108225

[R35] Robbins NM, Larimer P, Bourgeois JA, Lowenstein DH, Feb 2016. Number of patient-reported allergies helps distinguish epilepsy from psychogenic nonepileptic seizures. Epilepsy Behav. 55, 174–177. 26803428 10.1016/j.yebeh.2015.12.022 PMC4747833

[R36] Ronaldson A, Elton L, Jayakumar S, Jieman A, Halvorsrud K, Bhui K, 2020. Severe mental illness and health service utilisation for nonpsychiatric medical disorders: a systematic review and meta-analysis. PLoS Med. 17 (9), e1003284. 32925912 10.1371/journal.pmed.1003284 PMC7489517

[R37] Rosenberg SD, Lu W, Mueser KT, Jankowski MK, Cournos F, 2007. Correlates of adverse childhood events among adults with schizophrenia spectrum disorders. Psychiatr. Serv 58 (2), 245–253. 17287383 10.1176/ps.2007.58.2.245

[R38] Ross CA, Browning E, 2016. The relationship between catatonia and dissociation: a preliminary investigation. J. Trauma Dissociation 17 (4), 426–434. 26751346 10.1080/15299732.2015.1136858

[R39] Salinsky M, Storzbach D, Goy E, Kellogg M, Boudreau E, 2016. Health care utilization following diagnosis of psychogenic nonepileptic seizures. Epilepsy Behav. 60, 107–111, 2016/07/01/. 27206227 10.1016/j.yebeh.2016.04.007

[R40] Schäfer I, Fisher HL, Aderhold V, , 2012. Dissociative symptoms in patients with schizophrenia: relationships with childhood trauma and psychotic symptoms. Compr. Psychiatry 53 (4), 364–371. 21741038 10.1016/j.comppsych.2011.05.010

[R41] Stephen CD, Fung V, Lungu CI, Espay AJ, 2021. Assessment of emergency department and inpatient use and costs in adult and pediatric functional neurological disorders. JAMA Neurol. 78 (1), 88–101. 33104173 10.1001/jamaneurol.2020.3753 PMC7589058

[R42] Stubbs B, Mitchell AJ, De Hert M, Correll CU, Soundy A, Stroobants M, Vancampfort D, 2014. The prevalence and moderators of clinical pain in people with schizophrenia: a systematic review and large scale meta-analysis. Schizophr. Res 160 (1), 1–8, 2014/12/01/. 25458569 10.1016/j.schres.2014.10.017

[R43] survminer: Drawing Survival Curves using ’ggplot2 [computer program]. Version: R package version 0.4.9. Available at: https://CRAN.R-project.org/package=survminer.

[R44] Tan M, Pearce N, Tobias A, Cook MJ, D’Souza WJ, 2023. Influence of comorbidity on mortality in patients with epilepsy and psychogenic nonepileptic seizures. Epilepsia 64 (4), 1035–1045. 36740578 10.1111/epi.17532

[R45] Therneau T. A Package for Survival Analysis in R. Version: R package version 3.3–1. Available at: https://CRAN.R-project.org/package=survival.

[R46] Therneau TM, Grambsch PM, 2013. Modeling survival data: extending the cox model. Springer New York.

[R47] Trotta A, Murray R, Fisher H, 2015. The impact of childhood adversity on the persistence of psychotic symptoms: a systematic review and meta-analysis. Psychol. Med 45 (12), 2481–2498. 25903153 10.1017/S0033291715000574

[R48] Wu P, Gifford A, Meng X, , Nov 29 2019. Mapping ICD-10 and ICD-10-CM codes to phecodes: workflow development and initial evaluation. JMIR. Med. Inform 7 (4), e14325. 31553307 10.2196/14325 PMC6911227

[R49] Yates K, Lång U, Cederlöf M, Boland F, Taylor P, Cannon M, McNicholas F, DeVylder J, Kelleher I, Feb 1 2019. Association of Psychotic Experiences With Subsequent Risk of Suicidal Ideation, Suicide Attempts, and Suicide Deaths: A Systematic Review and Meta-analysis of Longitudinal Population Studies. JAMA Psychiatry 76 (2), 180–189. 30484818 10.1001/jamapsychiatry.2018.3514 PMC6439738

